# Biomolecular Adsorption on Nanomaterials: Combining Molecular Simulations with Machine Learning

**DOI:** 10.1021/acs.jcim.3c01606

**Published:** 2024-04-16

**Authors:** Marzieh Saeedimasine, Roja Rahmani, Alexander P. Lyubartsev

**Affiliations:** Department of Materials and Environmental Chemistry, Stockholm University, Stockholm SE-106 91, Sweden

## Abstract

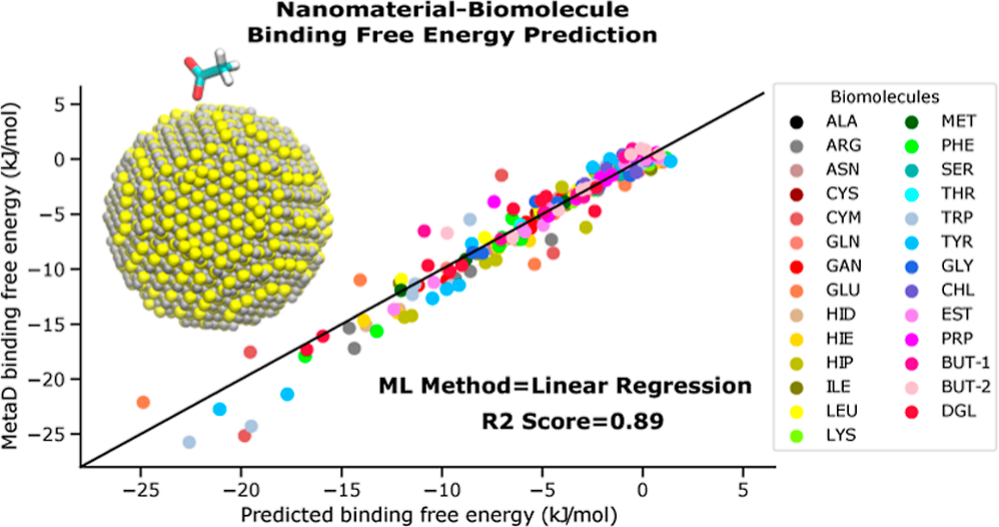

Adsorption free energies of 32 small biomolecules (amino acids side chains, fragments of lipids, and sugar molecules) on 33 different nanomaterials, computed by the molecular dynamics - metadynamics methodology, have been analyzed using statistical machine learning approaches. Multiple unsupervised learning algorithms (principal component analysis, agglomerative clustering, and K-means) as well as supervised linear and nonlinear regression algorithms (linear regression, AdaBoost ensemble learning, artificial neural network) have been applied. As a result, a small set of biomolecules has been identified, knowledge of adsorption free energies of which to a specific nanomaterial can be used to predict, within the developed machine learning model, adsorption free energies of other biomolecules. Furthermore, the methodology of grouping of nanomaterials according to their interactions with biomolecules has been presented.

## Introduction

Understanding of interactions between nanomaterials and biological matter is of primary importance for numerous biotechnology and biomedical applications, as well as for evaluation of nanomaterials eventual toxicity and ensuring their safety within the safe-by-design concept.^1−4^ Experimental characterization of the surface phenomena is difficult because of the small volume of the interface region relative to the bulk of the materials or solution. Molecular computer simulations, such as ab initio, classical atomistic, or coarse-grained molecular dynamics (MD), combined within consistent multiscale scheme,^5^ can provide deep insights into molecular phenomena at the material surface. However, such simulations are time-consuming, and it is practically impossible to carry out such MD simulations in a high-throughput manner for a large number of nanomaterial surfaces and their variations, in interaction with realistic biological environment which may include thousands of different biomolecules. Use of data-driven machine learning (ML) approaches becomes a natural choice when one needs to operate with a large amount of data.

ML techniques are nowadays growing tremendously in chemistry and materials science and have matured to become a powerful tool in nanomaterial design, characterization, and safety assessment.^6−8^ Quantitative structure–activity relationships (QSARs), supervised ML algorithms like linear regression (LR), support vector machine, artificial neural network (ANN), decision tree/random forest as well as unsupervised K-means and principal component analysis (PCA) have been applied to predict nanomaterials’ capabilities in areas of toxicity,^9,10^ adsorption and surface science,^11^ catalysis,^12^ and mechanical properties.^13^

Within data-driven and ML models, a set of descriptors characterizing a specific nanomaterial is used to predict the properties of the nanomaterials for their functionality and safety in the biological environment. Some widely used descriptors are general characteristics of the nanoparticles such as size, shape, surface charge, etc. It is less straightforward to find relevant descriptors which characterize the material itself and how it interacts with biomatter to distinguish between different types of materials such as metals, metal oxides, carbon-based nanomaterials, quantum dots, etc. It was suggested that adsorption free energies (called also binding free energies) of small molecules, representing typical fragments of biomolecules such as proteins and lipids, can be used as such descriptors, relevant for characterization of bionano interactions.^14^ Adsorption free energy is a well-defined physical property, it can be computed by molecular simulations and measured experimentally, and it is directly related to such molecular initiating events of toxicity pathways as biomembrane permeation or protein corona formation.^5^ A set of such adsorption free energies can be considered as a “biological fingerprint” of the nanomaterial that can be used in data-driven models for evaluation of functionality and safety of nanomaterials.^15^ In this line, Brinkmann et al^16^ analyzed adsorption affinity of microbial metabolites to carbon nanotubes and metal nanomaterials using QSAR and molecular dynamics - metadynamics simulations in relation to assessment of adverse effects of nanoparticles passing the gastrointestinal tract. Chen et al.^17^ used the biological surface adsorption index to cluster nanomaterials according to their surface physicochemical properties for biological/environmental predictions.

Adsorption free energies of small biomolecules such as amino acids and lipid fragments have been computed in molecular simulations for a number of nanomaterials in a variety of studies,^18−24^ and it was shown that such data can be further used to predict adsorption of proteins.^5^ QSAR models have been developed for protein adsorption on a nanoparticle surface which demonstrated that instead of fitting many parameters, only a few of the protein characteristics are actually important.^14^ It can be imperative to ask, what is the minimum set of molecules, adsorption free energies of which to a given nanosurface can be used to predict adsorption of an arbitrary biomolecule to this nanosurface and which can be further used to characterize the interaction of the nanomaterial with biomatter? Understanding these relationships would facilitate both computational and experimental characterization of nanomaterials with respect to interaction with biological environments since such characterization could be done with less amount of computational efforts or experiments.

Besides choosing the most relevant descriptors that directly affect the ML model’s interpretability, the model complexity is another question in using ML methods. Simple ML methods like LR and K-means can be applied to smaller data sets and easily understood while high-performance ANN needs larger training data sets and acts like a black box that may hinder users from identifying the weakness of the training model. Choosing the best ML algorithm to fulfill the accuracy, interpretability, and performance for modeling is challenging. The key point is to find an ML algorithm that compromises between complexity and accuracy in order to establish an accurate, efficient, and explicable model. In this work, three different ML algorithms, LR, decision tree-based ensemble learning, and one-hidden-layer neural network, have been applied to model biomolecule–surface adsorption free energy in order to find an optimized methodology that has a balance between model’s accuracy and performance.

In this work, we analyze data on adsorption free energies of over 30 small molecules representing various fragments of biomolecules to over 30 nanomaterial surfaces. We test several ML methods to develop predictive models of evaluation of adsorption free energies of small molecules to a specific nanomaterial from knowing the adsorption free energy of only a few selected molecules. Finally, we use the developed models to group nanomaterials in clusters such that nanomaterials in the same cluster have similar interactions with the biological environment and thus are expected to have similar biological responses.

## Material Models and Methods

### Overall Approach

The overall aim of our work is to identify a limited set of biomolecules, knowledge of adsorption free energies of which to a certain material can be used to predict adsorption free energies of other biomolecules to the same material that can be further used to classify, or to group, materials with respect to their interaction with biomatter. The developed approach includes the following steps:

- Data collection. Here, we collect numerical data on adsorption free energies of 32 small molecules to a set of 33 nanomaterials computed by MD simulations.
- Clustering of molecules. We use adsorption free energies data to cluster molecules into groups, which show similar interaction patterns with different materials, and select representatives of the groups.
- Regression models and their validation. We explore several methods to predict adsorption free energies of biomolecules from knowledge of adsorption free energies of a few molecules selected at the previous step.
- Grouping of nanomaterials. We use both the full data set of adsorption free energies and the predicted set of adsorption free energies to cluster nanomaterials into groups.

**Figure 1:**
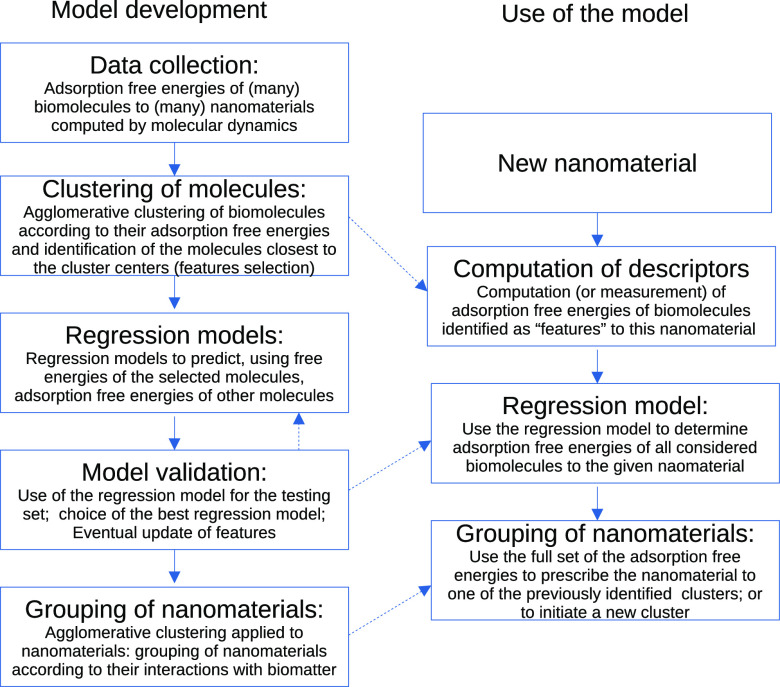
General scheme of the workflow of methods used during the model development and its intended use.

The workflow of the specific methods, during both the model development and its intended use, is illustrated in Figure 1. Detailed description of the algorithms used at each step is given below.

### Training Systems

**Figure 2:**
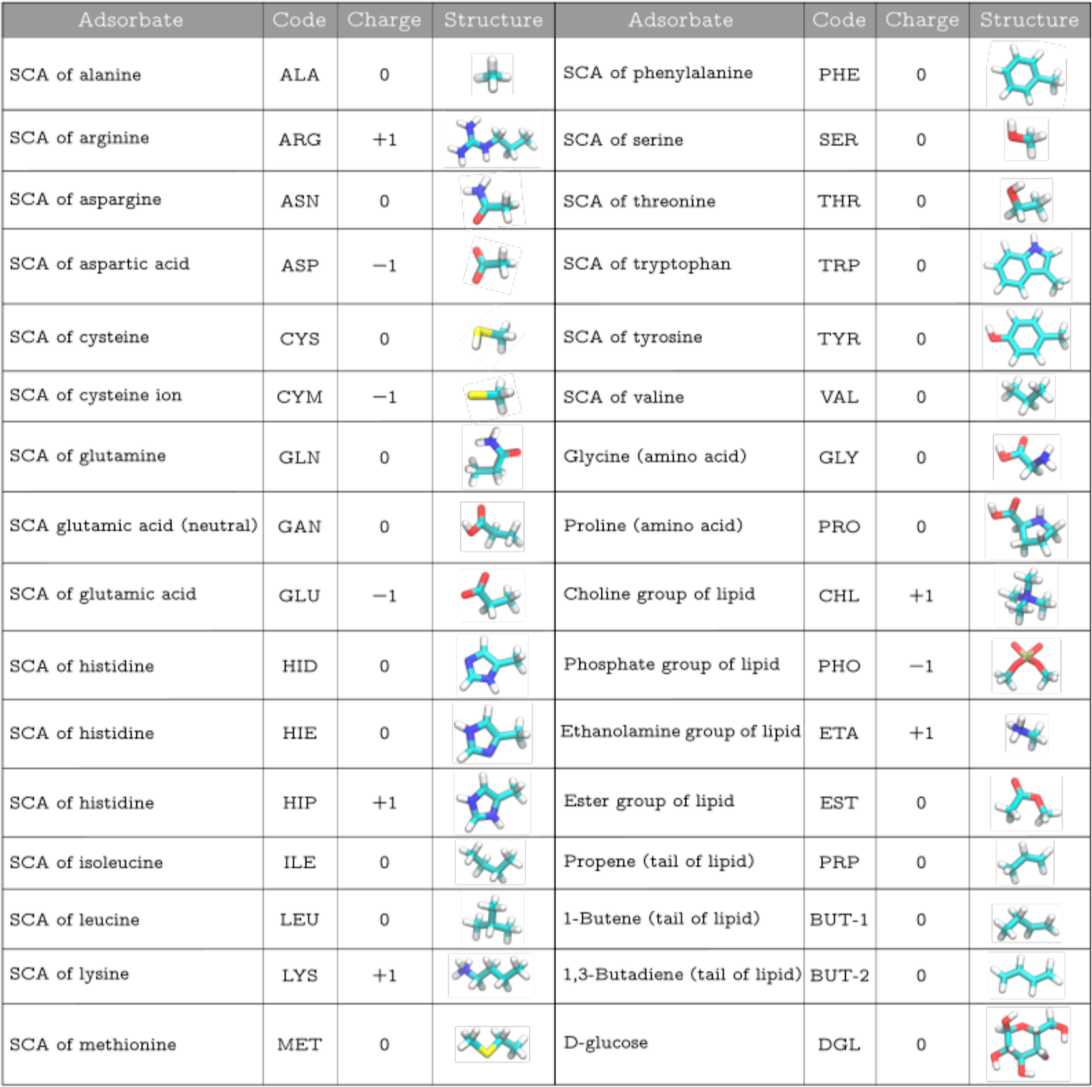
Chemical characterization of adsorbents.

Previously in our group, we computed adsorption free energies of a set of 29 biomolecules to a number of nanomaterials.^5,21,24,25^ These molecules include side-chain analogues of naturally occurring amino acids (except glycine and proline), full amino acids glycine and proline, protonated or unprotonated forms of some amino acids having p*K*_a_ values between 4 and 10, fragments of lipids, and d-glucose. In order to make the data set more extensive and more suitable for ML analysis, in this work we carried out additional free energy computations. Thus, we added three additional molecules representing fragments of unsaturated lipids and computed adsorption free energies of these molecules to all considered in the previous papers’ nanomaterials. The selected set of molecules represents constituting fragments of the most essential biomolecules: proteins, lipids, and glucans and thus cover a major part of the biochemical molecular space. Furthermore, we computed adsorption free energies of the extended set of 32 molecules to several other nanomaterials, not considered in the previous studies. The whole set of considered biomolecules, together with their notation through the text, is presented in Figure 2.

As nanomaterial surfaces, we in previous studies considered carbon-based nanomaterials including unstructured amorphous carbon, pristine graphene and its derivatives (such as few-layer graphene, graphene oxide, and reduced graphene oxide), pristine carbon nanotubes (CNTs), and those functionalized with −OH, −COOH, −COO^–^, −NH_2_, and –NH_3_^+^ groups in two different concentrations: low and high. The low value corresponds to typical experimental conditions (a few wt %), while the high value is the maximum concentration allowed while keeping an intact CNT. Other nanomaterials for which adsorption free energy data are available from previous studies include several types of metal oxides: titanium dioxide surfaces with lower surface energy [TiO_2_-rutile (110) and (100) as well as TiO_2_-anatase (101) and (100)], silicon dioxide (SiO_2_ in quartz and amorphous form), iron oxide (Fe_2_O_3_(001) oxygen-terminated surface), and ZnS semiconductor in several modifications: pristine ZnS(110) surface, poly methyl methacrylate (PMMA)-coated ZnS(110) surface, and spherical ZnS nanoparticle of 5 nm diameter. The surface of metal oxide nanomaterials was modified by setting hydroxyl groups and protonated oxygens at a specified fraction of the surface sites to be consistent with experimental zeta potential, while the rest of the surface sites had molecularly bound water in the BOND model or were left free.^26^ To extend the data set, in this work we carried out computations for several additional nanomaterials: two zinc oxide surfaces [ZnO(101̅0) and ZnO(12̅10)], cadmium selenide (CdSe), and additional TiO_2_ surfaces without molecularly bound water. The extended set of nanomaterials thus includes representatives of the most important classes of nanomaterials: carbon-based, metal oxides, semiconductors (quantum dots), with emphasis on the most used nanomaterials (CNTs, graphene, TiO_2_) which are presented in several variations. Furthermore, our choice includes both hydrophobic and hydrophilic nanomaterials as well as charged and uncharged surfaces. The whole set of considered nanomaterials, together with their notation through the text and references to the force field parametrization and to the studies on adsorption free energy computation, is given in Table 1. We also prolong some previous computations for CNT nanomaterials in order to reduce standard deviation errors.

**Table 1:** Chemical Characterization of Considered Nanomaterials, Including Reference to the Force Field (FF-Ref), and Reference to the Work Where Adsorption Free Energies Were Computed (Ads-Ref)

description	Code	chemical composition	FF-ref	Ads-ref
amorphous carbon	C-AM-1	C_1944_	(27,28)	(21)
amorphous carbon	C-AM-2	C_1944_	(27,28)	(21)
amorphous carbon	C-AM-3	C_1944_	(27,28)	(21)
Graphene	GR	C_416_	(28)	(21)
bilayer graphene	bi-GR	C_1664_	(28)	(21)
trilayer graphene	tri-GR	C_2496_	(28)	(21)
graphene oxide	GO	C_336_(OH)_86_(O)_39_H_54_	(28)	(21)
reduced graphene oxide	rGO	C_336_(OH)_20_(O)_14_H_57_	(28)	(21)
CNT (11,11)	CNT	C_660_	(28)	(21)
CNT-OH (3.9 wt %)	CNT-OH-low	C_660_(OH)_19_	(28)	(21)
CNT-OH (14 wt %)	CNT-OH-high	C_660_(OH)_76_	(28)	(21)
CNT-COOH (2.8 wt %)	CNT-COOH-low	C_660_(COOH)_5_	(28)	(21)
CNT-COOH (30 wt %)	CNT-COOH-high	C_660_(COOH)_77_	(28)	(21)
CNT-COO-€ (2.7 wt %)	CNT-COO—low	C_660_(COO^–^)_5_	(28)	(21)
CNT-COO^–^ (10 wt %)	CNT-COO—high	C_660_(COO^–^)_20_	(28)	(21)
CNT-NH_2_ (2 wt %)	CNT-NH_2_-low	C_660_(NH_2_)_10_	(28)	(21)
CNT-NH_2_ (13.8 wt %)	CNT-NH_2_-high	C_660_(NH_2_)_79_	(28)	(21)
CNT-NH_3_^+^ (2.1 wt %)	CNT-NH_3_^+^-low	C_660_(NH_3_^+^)_10_	(28)	(21)
CNT-NH_3_^+^ (4.1 wt %)	CNT-NH_3_^+^-high	C_660_(NH_3_^+^)_20_	(28)	(21)
TiO_2_-ana(101)-NB	TiO_2_-ana(101)	(TiO_2_)_2340_(OH)_78_	(5)	this work
TiO_2_-ana(101)-BOND	TiO_2_-ana(101)-B	(TiO_2_)_768_(OH)_14_(H_2_O)_82_	(5)	(5)
TiO_2_-ana(100)-BOND	TiO_2_-ana(100)-B	(TiO_2_)_672_(OH)_28_(H_2_O)_68_	(5)	(5)
TiO_2_-rut(100)-BOND	TiO_2_-rut(100)-B	(TiO_2_)770(OH)28(H_2_O)112	(5)	(5)
TiO_2_-rut(110)-BOND	TiO_2_-rut(110)-B	(TiO_2_)800(OH)30(H_2_O)70	(5)	(5)
ZnO(101̅20)	ZnO(101̅0)	(ZnO)_864_(OH)_63_H_63_	(26)	this work
ZnO(12̅10)	ZnO(12̅10)	(ZnO)_1152_(OH)_90_H_90_	(26)	this work
ZnS(110)	ZnS(110)	(ZnS)_1344_	(29)	(24)
PMMA-coated-ZnS(110)	ZnS(110)-coated	(ZnS)_1344_([C_5_H_8_O_2_]_3_)_70_	(29)	(24)
ZnS nanoparticle	ZnS-NP	(ZnS)_1800_	(29)	(24)
SiO_2_-Q4 (quartz)	SiO_2_-Q4	(SiO_2_)_672_	(30)	(25)
SiO_2_-Q2 (amorphous)	SiO_2_-Q2	(SiO_2_)_504_(OH)_112_H_112_	(30)	(25)
Fe_2_O_3_(001)-*O*-terminated	Fe_2_O_3_(001)	(Fe_2_O_3_)_1920_(OH)_240_H_240_	(25)	(25)
CdSe	CdSe	(CdSe)_288_	(31)	this work

## Methods

### Adsorption Free Energy Calculations

All adsorption free energies analyzed in this work (see full account of the studied systems in the previous section) were computed by advanced sampling metadynamics simulations implemented in the PLUMED module v 2.7^32^ to Gromacs 2020 or 2021 software.^33^ The detailed description of the algorithms and methods to ensure convergence of the simulations and analyze uncertainty are given in our previous publications.^5,21,24^ Complementary adsorption free energy computations of this work were carried out according to the same methodology. Here, we recapitulate the basic features and parameters of these computations.

The *z*-component of the distance between the surface of the nanomaterial (atoms in the outermost layer) and the center of mass (COM) of the adsorbate, called surface separation distance (SSD), was used as a collective variable except for the ZnS spherical nanoparticle and for amorphous carbon, where the SSD was determined as the minimum distance between the COM of the adsorbate and a surface atom because the former definition of SSD is not accurate enough for a rough surface. Each simulation was started by placing the adsorbate molecule outside the nanomaterial and filling the rest of the simulation box with water. For charged nanosurfaces, the system was neutralized by adding appropriate number of Na^+^ or Cl^–^ ions, additional ions were added to provide a salt concentration at the physiologic conditions (0.15 M). The systems were initially equilibrated by running nonbiased simulation in semianisotropic *NPT* ensemble. The last configuration of that simulation was used as a starting point of the metadynamics simulation, which was run in the *NVT* ensemble with a constant Gaussian height of 0.001 kJ/mol deposited every 500 steps for at least 600 ns. The first 50 ns of the simulation was excluded from the analysis. The production part was prolonged for some combinations of sorbent—nanomaterial up to 1000 ns to improve statistical uncertainty. The potential of mean force (PMF) *W*(*s*) was calculated by integration over the average force (⟨*F*(*s*)⟩) acting on the adsorbent molecule at each SSD as follows
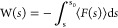
1where *s*_0_ is taken on a large distance from the surface where average force is negligibly small. Adsorption free energy is then obtained from the PMF by
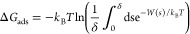
2where *k*_B_*T* is the product of the Boltzmann constant and the absolute temperature and δ is the adsorption layer thickness which was set to 0.8 nm in our calculations. We call this quantity MetaD adsorption free energy in the rest of the text. The PMF was evaluated during each 100 ns window of the production part of the simulation. The variance of the PMF over windows was used to estimate minimum and maximum values of the PMF at each distance point and was then used to determine maximum and minimum values of the adsorption free energies. In most cases, the statistical uncertainty of free energies was found within 0.5 kJ/mol.

Other important simulation parameters are listed below. A V-rescale thermostat^34^ with a relaxation time of 1 ps was used to keep temperature *T* = 300 K constant. Particle-mesh Ewald summation for electrostatic and Lennard-Jones interactions^35^ was employed with a grid spacing of 0.12 nm. All bonds to hydrogen atoms were constrained by applying LINCS algorithm.^36^

In all computations, the biomolecules were described by the general amber force field (GAFF) (version 2.11) with parameters generated by running antechamber^37^ via acpype.^38^ The TIP3P model^39^ was used for water molecules. Carbon-based nanomaterials were modeled with the GAFF parameters. For ZnO and TiO_2_ force field parameters, compatible with biomolecular force fields, have been derived in previous studies from ab initio MD simulations.^5,26^ References for force field parameters of each nanomaterial can be found in Table 1. More computational details for each specific system can be found in references given in Table 1. The whole data set of adsorption free energies used in this work, including estimated uncertainties, is provided in the data archive as a part of the Supporting Information.

### Machine Learning Methods

For clustering of biomolecules into groups and selection of group representatives, we have used PCA, agglomerative clustering,^40^ and K-means.^41^ PCA was used for linear dimensionality reduction of the data using singular value decomposition^42^ in order to get indication on the optimal number of clusters. The agglomerative clustering and K-means algorithms were used to create the clustering models. Euclidean distance was used to calculate the distance between instances, and the Ward method was used to compute the distance between the clusters (linkage distance).

After grouping biomolecules to the identified clusters and selecting cluster representatives, we have used several regression methods to develop models of prediction of the free energies of biomolecules from knowing the free energies of only several selected molecules. In other words, adsorption free energies of selected molecules were “features”, while adsorption free energies of other molecules were “responses”. The LR algorithm was employed to create a linear model. LR was chosen for its simplicity and straightforward implementation. To improve and boost regression modeling, an ensemble learning method of AdaBoost (AdaBoostRegressor)^43^ was also tested. AdaBoost was selected due to its capability to enhance predictive performance by sequentially combining weak learners and assigning higher weights to misclassified instances in subsequent iterations, thereby focusing on areas where model performance is weaker. Here, the decision tree regressor^44^ was employed as a weak learner with a max depth of three, and we used 50 boosting iterations with linear loss function. Finally, we applied ANN because of its capacity to capture intricate nonlinear relationships in data, making it suitable for scenarios where the underlying patterns are complex and not easily captured by simpler models. In the ANN model, multilayer perceptron regressor (MLPRegressor)^45^ with tanh activation function was used, together with L2-regularization scheme to prevent overfitting of the training data and Adam stochastic gradient-based optimizer for weight optimization. Testing of these three regression methods in predictions of adsorption free energies allows for an exploration of the trade-offs between model complexity and predictive performance. Linear regression provides interpretability but may lack accuracy in capturing complex relationships. AdaBoost may improve performance through boosting but may be sensitive to noisy data, leading to overfitting. ANN excels in capturing nonlinear patterns but can be computationally intensive and may require larger data sets.

For each regression method, train-test splitting was created by random splitting of the whole data set into two parts, with 70% of the nanomaterials as the training set and 30% as the testing set. ShuffleSplit was also used to make random permutations resulting in 10 different splittings of the whole data set. Scikit-learn^46^ library of Python was used to implement the used methods and algorithms.

To evaluate the accuracy of the model, the *R*^2^ score (coefficient of determination) and the mean absolute error (MAE) have been calculated as follows
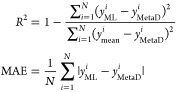
3where *y*_ML_ and *y*_MetaD_ are predicted by the ML model and computed by metadynamics free energies, respectively, and *y*_mean_ is the average of MetaD free energies in the considered set of data points. The standard deviation of the *R*^2^ score and MAE were estimated by calculating these values for 10 different iterations of train-test splitting on the entire data set.

## Results and Discussion

### Dimensionality Reduction

Computations of biomolecule–surface adsorption free energy for many (32 in our case) biomolecules to provide nanomaterial biological fingerprint are computationally demanding, and we want to identify a limited set of biomolecules that determine adsorption behavior of other molecules. We first tested the PCA in order to project the high-dimensional data to a lower dimensional space and quantify the variance of data along each principal component. The maximum likelihood estimate (MLE) algorithm^42^ is used to guess the number of principal components. The amount of variance explained by each of the principal components is shown in Figure S1 of Supporting Information. Three major principal components can be identified with a significant variance while the explained variances along the other principal components are negligible.

In order to quantify the direction of each principal axes in the new feature space, we calculated the absolute value of eigenvectors along the original features (32 biomolecules) for three main principal components, which are shown in Figure S2 of Supporting Information. For the first principal component with maximum variance, larger eigenvalues are found along the aromatic or cyclic biomolecules like TYR, TRP, HID, PHE, ARG, DGL, and PRO. In the second principal component, negatively charged residues ASP, GLU, CYM, and PHO contribute significantly while positive and polar species contribute significantly to the third component. Although PCA characterizes the number of main principal components to reduce the features in modeling, it cannot identify the most relevant ones, that is do feature selection.

In the following, we apply clustering methods to assign biomolecules to different clusters based on the similarity/dissimilarity of adsorption free energies in order to select relevant biomolecules (features) in biomolecule–surface adsorption free energy modeling.

### Clustering of Biomolecules

**Figure 3:**
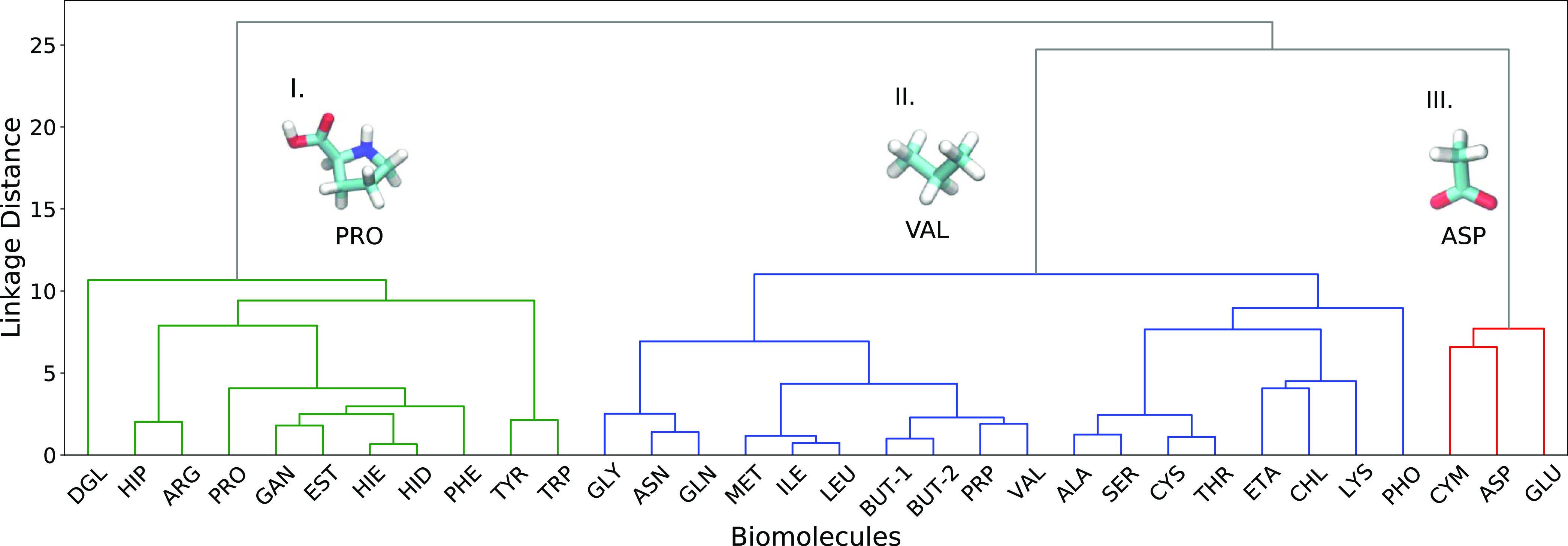
Dendrogram of biomolecules obtained by the agglomerative clustering according to their binding free energies to nanomaterials. Division on three major clusters (referred in the text as groups I, II, and III) are highlighted by different colors. Representative molecules of each group determined by the closest distance to the cluster centers are shown.

In order to select biomolecules (features) for biomolecule–surface adsorption free energy modeling, unsupervised ML clustering methods have been used to classify biomolecules into groups (clusters) with similar attributes (interaction) to different nanomaterials. Here, we employed the hierarchical agglomerative clustering algorithm to cluster biomolecules based on a matrix of root mean squared distances of MetaD adsorption free energy values on different nanomaterials. This approach results in a cluster hierarchy of biomolecules with similar behavior in the interaction with the nanomaterials. Cluster tree for clustering of biomolecules is shown in Figure 3. By considering sufficiently large linkage distance in the dendrogram of biomolecules clustering, we can observe three distinct clusters (highlighted in Figure 3 by different colors) of biomolecules as follows: (I) prevailing aromatic biomolecules, (III) small-sized biomolecules with a negative charge, and (II) rest of biomolecules. This grouping is well correlated with the results of PCA.

Group (I) consists of aromatic or cyclic biomolecules like TYR, TRP, HID, PHE, ARG, DGL, and PRO which distinguish from others in that they interact strongly with hydrophobic carbon-based nanomaterials such as CNTs or graphene due to favorable π–π interactions.^21^ Arginine is not a cyclic molecule, but its guanidinium group has sp^2^ hybridized atoms which forms a quasi-aromatic structure that can engage in π–π stacking interactions. Group (III) (shown in red in Figure 3) consists of ASP, GLU, and CYM which are small-sized biomolecules with a negative charge. This group interacts selectively with nanomaterials with positive surface charges or with surface-exposed positively charged atoms of metal oxides due to charge–charge interactions.^24,26^ Group (II) consists of many molecules that can be further divided into smaller groups by lowering the linkage distance. It is notable that hydrogen bonding molecules are not forming a group but appear mostly in group II together with small hydrophobic and some bulky charged molecules. Another remarkable result is that the negatively charged phosphate residue (PHO) does not appear in group III with other negatively charged molecules. A possible explanation can be that in the phosphate, negative charge is distributed over 4 oxygen atoms, two of which are screened by the methyl groups, thus it behaves as a more “bulky” ion. Anionic molecules of group III have either carboxylic charged group COO–, or sulfur, with a strong negative charge localized at the edge of the molecule. These groups can interact strongly with positively charged metal sites at the surface of metal oxide or semiconductor materials, while the more bulky PHO residue cannot displace water molecules typically bound to such sites, and generally, PHO shows weaker binding to polar surfaces, more similar to other molecules of the group II.

We have also tried a nonhierarchical clustering technique of K-means. This method partitions all data points into a predefined number of sets to minimize the within-cluster variance and maximize the between-cluster variance. Figure S3 of Supporting Information shows “within-cluster” sum of square distances (called inertia) as a function of the number of tested clusters. As the number of clusters increases, the inertia generally decreases. The “elbow” point in the curve, located at *k* = 3, indicates the optimal number of clusters. Thus, all tested methods, PCA analysis, agglomerating clustering, and *k*-means, points *k* = 3 as the optimal number of clusters. Note further that for *k* = 3 the *K*-means clustering classifies biomolecules in the same groups as agglomerative clustering.

In order to select a set of biomolecules (features) for biomolecule–surface adsorption free energy modeling, we identified within the hierarchical agglomerative clustering algorithm one representative biomolecule for each cluster by calculating the distance of the biomolecule to the center of the cluster and choosing the biomolecule with the smallest distance to the center of the cluster. PRO, VAL, and ASP have been selected as features according to this principle (see Table S1 of Supporting Information showing all distances to the cluster centers). By selecting these biomolecules, we aim to predict other biomolecular adsorption free energies as a function of the free energies of the three selected biomolecules. While the choice of the representatives was done by a formal criteria to be closest to the cluster center, the choice of proline as representative of group I is intriguing, taking in mind that this residue differs from other amino acids by binding to the protein backbone by two bonds. On the other hand, proline is taken separately has a ring structure similar to other molecules of the group. It was also noted previously that proline has some specificity in interactions with nanoparticles. Thus, Ranjan et al.^47^ studied interaction of TiO_2_ with different proteins and found that titanium dioxide nanoparticles frequently interacted with proline, lysine, and leucine within proteins, exhibiting a stronger binding affinity with proteins that contain these particular amino acids. Zuo et al.^48^ also showed the importance of proline-rich motifs in protein in interaction with carbon-based nanoparticles. These findings give indication that the proline residue might play a specific role in the interactions between biomolecules and nanomaterials.

### Machine Learning Prediction Models

**Figure 4:**
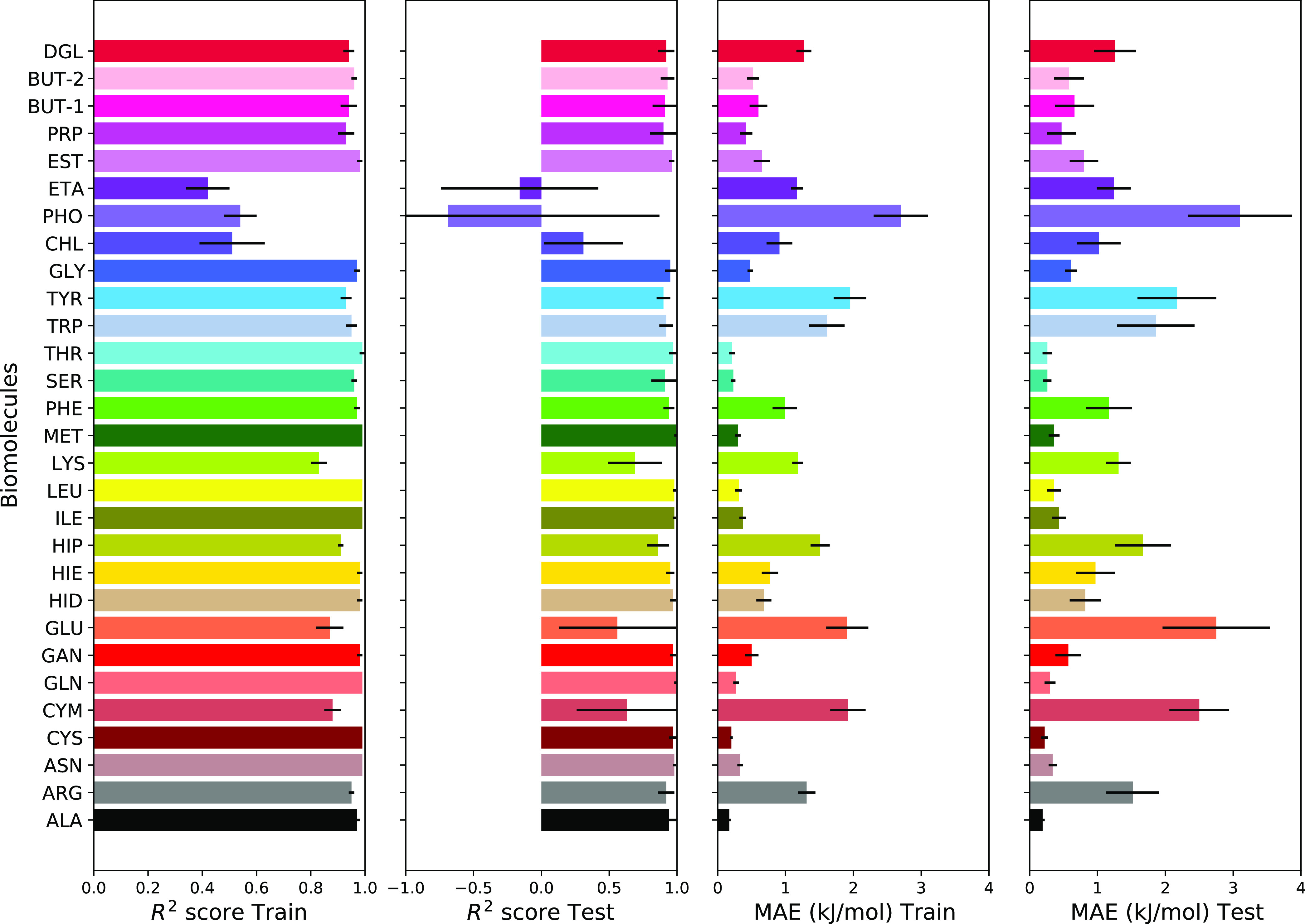
*R*^2^ score and mean absolute error for LR modeling of biomolecule–surface adsorption free energy with adsorption free energies of ASP, VAL, and PRO biomolecules are used as nanomaterials’ features.

We first tested the LR algorithm to fit the MetaD adsorption free energy. Adsorption free energies of three selected biomolecules (PRO, VAL, and ASP) on different nanomaterials have been used as input data (features) for modeling of free energy of the other 29 biomolecules. The training was made over the training set of nanomaterials, which included 70% of randomly chosen (23 of 33) nanomaterials. From the selected training set, a model was derived which predicted adsorption free energies of 29 “other” molecules from the free energies of 3 selected, and this model was then used to predict the free energies of 29 “other” molecules in the testing set of nanomaterials (which was not included in training). To quantify the accuracy of the resulted model, the *R*^2^ score and MAE of the adsorption free energy of each molecule to nanomaterials of both training and testing data sets have been calculated and averaged over 10 different random splittings between training and testing data sets. The results are listed in Figure 4. For most of the molecules, LR modeling fits the computed by MetaD results well, with an *R*^2^ score value of about 0.9 for both training and testing sets, and with an MAE under 2 kJ/mol. Although the LR algorithm can reasonably model adsorption free energy for most of the considered molecules, our result for PHO and ETA (and to a lesser degree for CHL) shows *R*^2^ score below 0.5 for the training set and a negative *R*^2^ score for the testing set. This draws the average *R*^2^ score for the testing down to 0.79, which is not satisfactory. Analysis of data for PHO and ETA shows that a large discrepancy comes from strong adsorption of PHO (negatively charged phosphate group) on NH_3_^+^-functionalized CNTs and adsorption of ETA (positively charged ethanolamine group) on COO^–^-functionalized CNTs. Charged molecules and charged surfaces are relatively poorly presented in the data set which may be the reason for poor reproduction of their adsorption free energy in the LR model.

**Figure 5:**
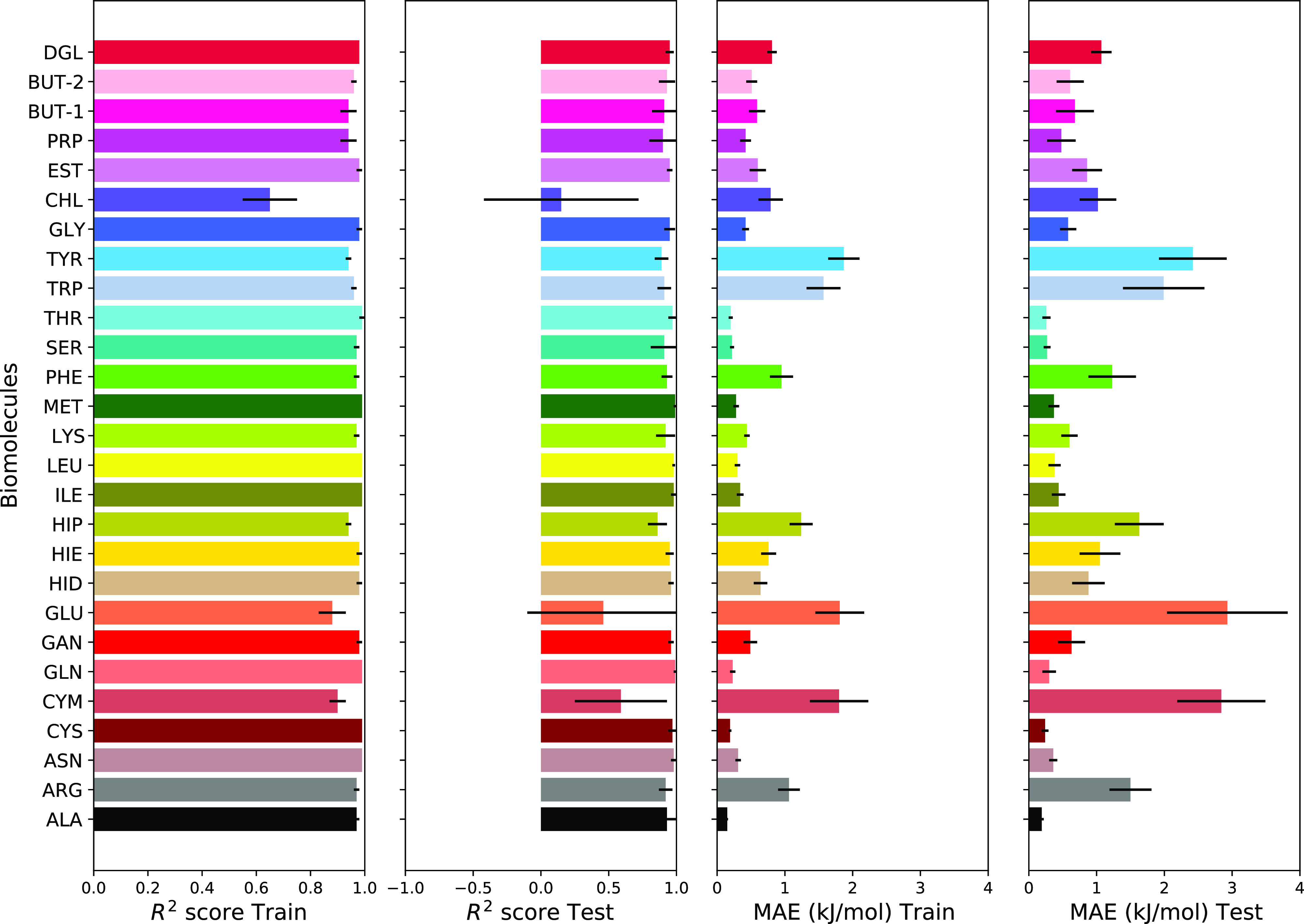
*R*^2^ score and MAE for LR modeling of biomolecule–surface adsorption free energy by adding free energies of PHO and ETA biomolecules (besides ASP, VAL, and PRO) as nanomaterials’ features.

**Figure 6:**
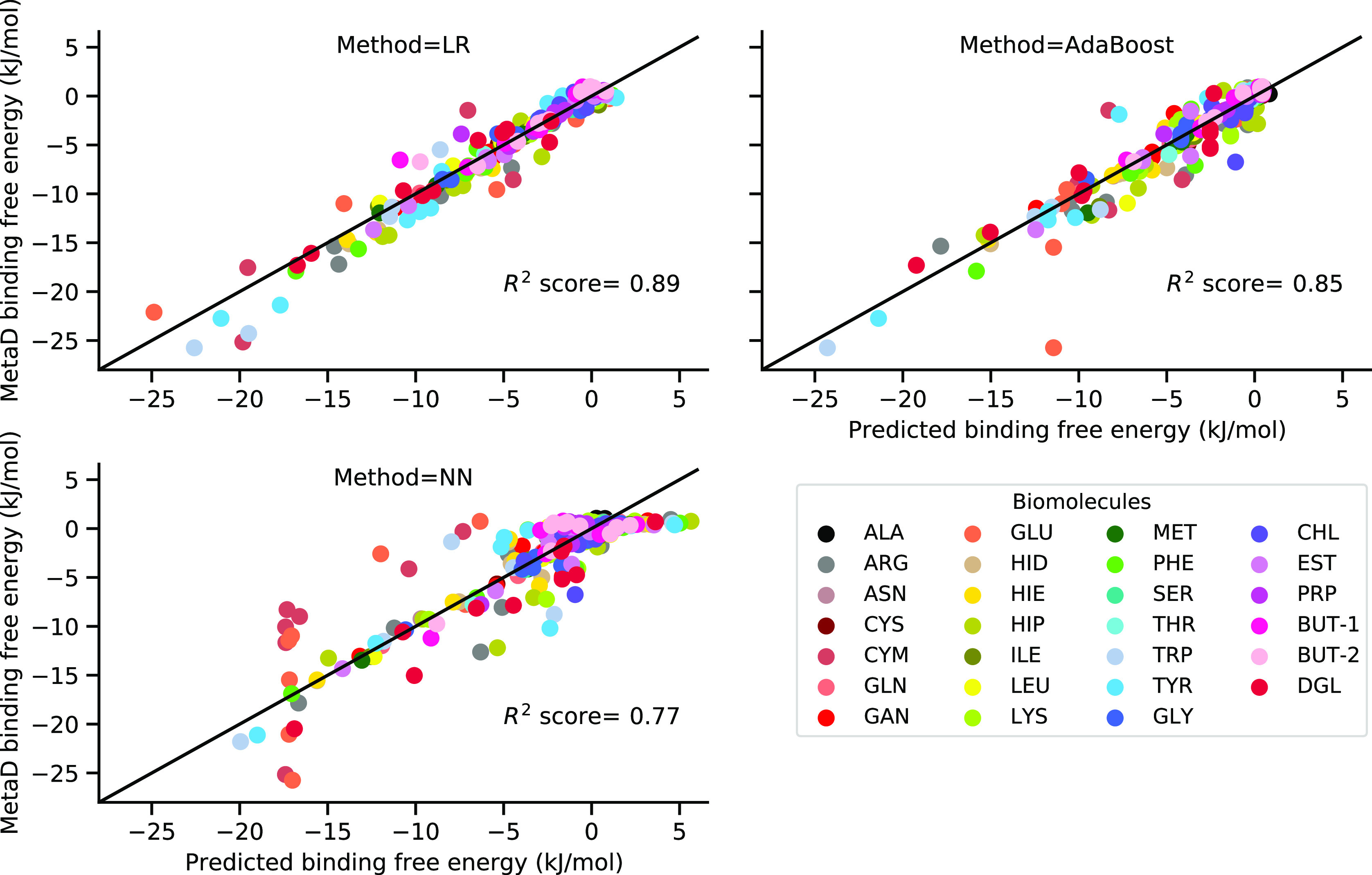
Predicted vs computed by MetaD biomolecule–surface adsorption free energies for testing data set by applying different ML algorithms. Data for one specific splitting between training and testing data sets with *R*^2^ score close to the average *R*^2^ score are shown.

To improve accuracy in the adsorption free energy prediction, we can increase the number of features feeding into the LR modeling or apply other ML algorithms. First, we modified our LR model by adding PHO and ETA adsorption free energies as features into the training data set. The performance of our modified LR model with five features is shown in Figure 5. *R*^2^ score for both training and testing data sets is improved and in many cases is close to 1. Predicted by LR modeling adsorption free energies with respect to the MetaD results are shown in Figure 6 with an average *R*^2^ score of 0.88 for all biomolecules (one of ten examples with the *R*^2^ score close to average is shown in Figure 6). The *R*^2^ and MAE results averaged over 10 different splittings between training and testing data sets are gathered in Table 2.

**Table 2:** ML Models (LR, AdaBoost, and NN) Performance Expressed by the *R*^2^ Score and MAE Taken over all Biomolecules Except the Selected Ones and Nanomaterials for the Training or Testing Sets and Averaged over 10 Different Divisions between Training and Testing Data Sets^a^

ML methods	*R*_Train_^2^	*R*_Test_^2^	MAE_Train_ (kJ/mol)	MAE_Test_ (kJ/mol)
LR (3)	0.91 ± 0.02	0.79 ± 0.15	0.88 ± 0.12	1.03 ± 0.26
LR (5)	0.95 ± 0.01	0.88 ± 0.09	0.70 ± 0.11	0.96 ± 0.24
AdaBoost (3)	1.0 ± 0.01	0.68 ± 0.39	0.06 ± 0.03	1.08 ± 0.39
AdaBoost (5)	1.0 ± 0.01	0.86 ± 0.10	0.05 ± 0.02	0.99 ± 0.33
NN (3)	0.92 ± 0.01	0.74 ± 0.17	0.84 ± 0.12	1.39 ± 0.38
NN (5)	0.95 ± 0.01	0.77 ± 0.19	0.58 ± 0.11	1.44 ± 0.38

a The number of selected biomolecules as features is shown in parentheses.

Next, we tried to improve the performance of ML modeling of biomolecule–surface adsorption free energy by applying other regression algorithms and comparing with LR modeling. We applied an ensemble ML technique^49^ in which predictions from multiple ML models are combined in order to bring better predictive results (in terms of accuracy and performance). An advantage of ensemble learning is that it allows users to combine simpler classifiers with more control over feature selection to increase the interpretability and efficiency of the combined methods. Bootstrap aggregation (bagging), boosting, and stacking are three popular ensemble learning techniques.^49^ Here, we applied the AdaBoost boosting algorithm in which the decision tree regressor is used as the base estimator (weak learner). In this method, first, a decision tree regressor fits on the original data set and then additional copies of the regressor (maximum 50 estimators) are applied, and the weights of instances are adjusted according to the error of the previous estimator. As such, subsequent regressors focus more on difficult cases in order to increase the accuracy of modeling. The performance of AdaBoost modeling of biomolecule–surface adsorption free energy is shown in Figure S4 of Supporting Information by considering three selected biomolecules (PRO, VAL, and ASP) and in Figure S5 for five selected biomolecules (PRO, VAL, ASP, PHO, and ETA) as modeling features, as well as in Table 2. *R*^2^ score of AdaBoost modeling is close to 1 and MAE is reduced to below 0.1 kJ/mol in the training data set, while not much improvement is observed for the testing data set compared to the LR modeling. The predicted AdaBoost adsorption free energy for the testing set shows an average *R*^2^ score of 0.68 and 0.86 for models with 3 and 5 features, respectively, which is lower compared to the LR modeling. A large difference in *R*^2^ score between training and testing data sets can be due to overfitting, when AdaBoost, due to a higher flexibility almost perfectly fits the training data but is unable to correctly reproduce testing data.

We have also tested whether a nonlinear neural network could improve prediction of free energy. Since our data set is small, we implemented one-hidden-layer NN with 10 nodes using the tanh activation function. The performance of NN modeling of biomolecular adsorption free energy using 3 selected biomolecules is shown in Figure S6, while result for the extended set of 5 molecules is shown in Figure S7 of Supporting Information, as well as in Table 2. An example of the predicted adsorption free energy with respect to the MetaD adsorption free energy is also shown in Figure 6. While providing similar to the LR model quality of prediction for the training data set, the *R*^2^ score for the testing data set in the NN model, 0.74 and 0.77 for models with 3 and 5 features, respectively, appeared to be lower compared to the LR model.

Summarizing results obtained within the three considered ML models (LR, AdaBoost, and NN), we can conclude that simple LR performs better than the two others for the prediction of biomolecular adsorption free energy, providing a high average *R*^2^ score of 0.88 and MAE within 1 kJ/mol for the testing data set. Coefficients of this best-performing LR model are given in Table S2 of Supporting Information. Predictions are less good for a few charged molecules (PHO, ETA, and CHL); however, more elaborated AdaBoost ensemble and NN algorithms do not help to improve the prediction of binding free energy for these molecules (see Figures 4, and S4 and S6 of Supporting Information), which can be due to the limited number of studied nanomaterials in our data set interacting selectively with PHO and ETA biomolecules.

As an additional test of the developed prediction algorithm, we have made computations removing randomly selected 30% of nanomaterials already on the stage of clustering of biomolecules and then followed the same methodology as described in the Methods section. We repeated clustering procedure for the limited (70%) set of the nanomaterials (see result in Figure S8), found the molecules which were closest to the cluster centers, trained the model by considering the same selected set of 70% of nanomaterials, and used the remaining 30% of nanomaterials as a testing set. In this scheme, the model has never seen any data from the 30% of nanomaterials of the testing set, neither at the clustering stage nor in the training stage. Still the results for the testing set presented in Supporting Information (Figures S9 and S10) are very similar to the original approach described in the Methods section (Figures 4,5), with average *R*^2^ score of 0.86 and MAE 0.93 kJ/mol for the testing set and LR with 5 features. This test gives confidence that no leaking of data occurs during clustering of biomolecules when the full data set is used.

### Clustering of Nanomaterials

**Figure 7:**
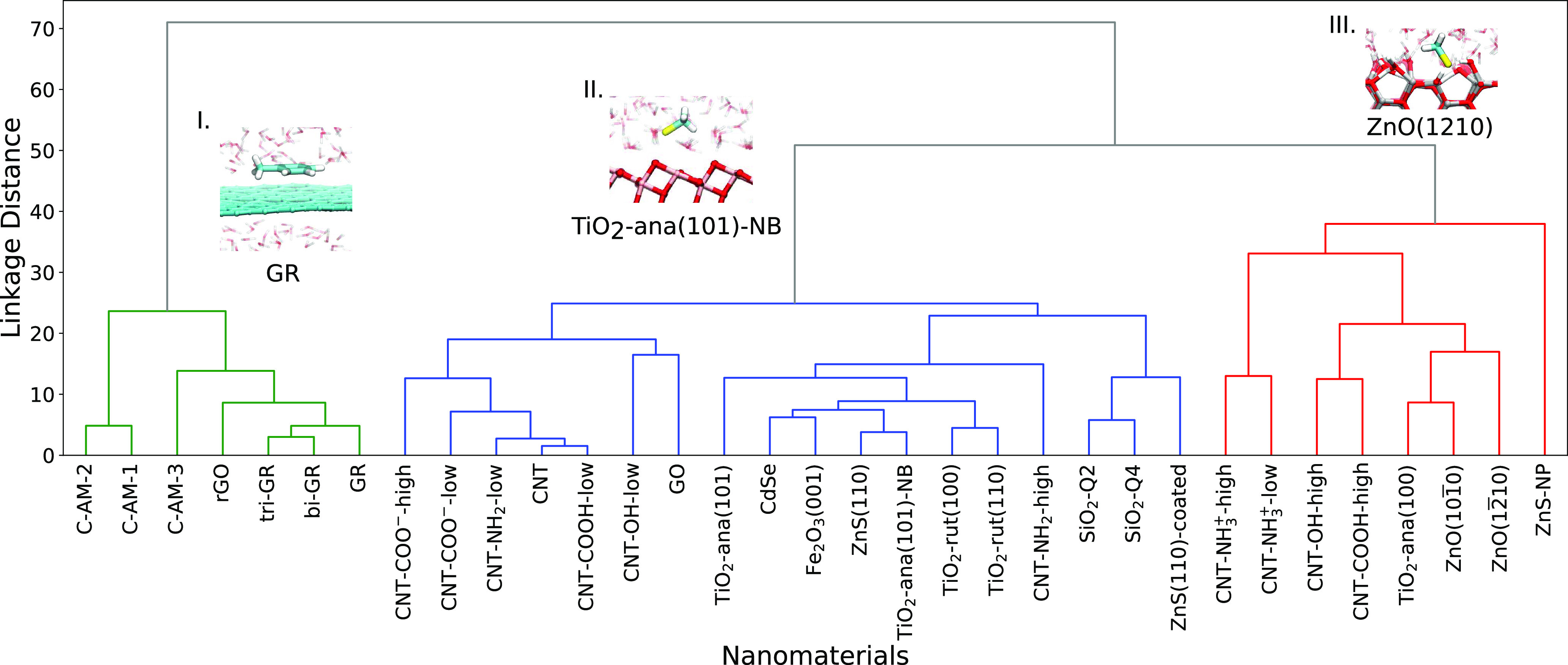
Dendrogram of nanomaterials’ agglomerative clustering obtained from the full adsorption free energies data set. Insets show typical adsorption modes of biomolecules.

**Figure 8:**
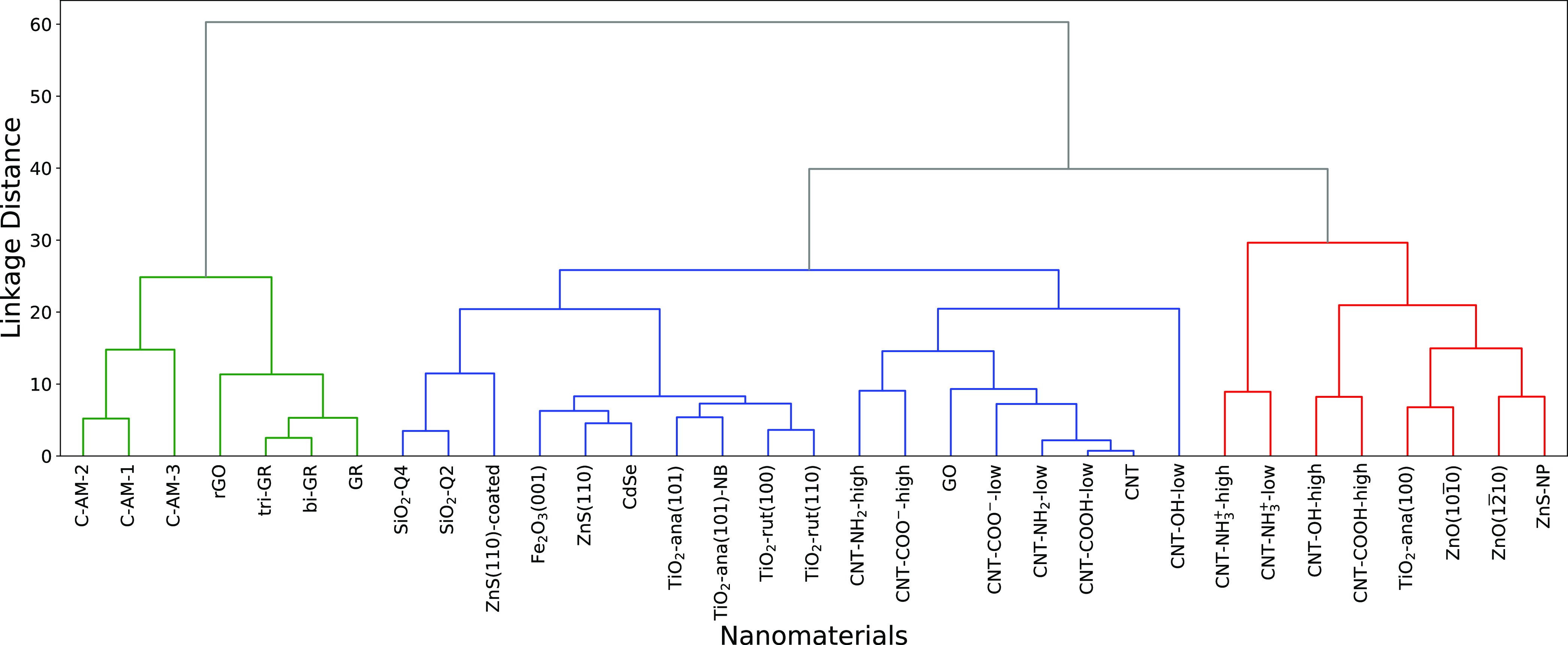
Dendrogram of nanomaterials’ agglomerative clustering obtained from the adsorption free energies predicted by the LR modeling with five features.

Finally, we used both the full data set of the adsorption free energies and predicted set of adsorption free energies to cluster nanomaterials into groups according to their interaction with biomolecules. Agglomerative clustering was applied for this purpose. The dendrogram of nanomaterials’ clustering using the full data set of adsorption free energies is shown in Figure 7 while the dendrogram built from the predicted adsorption free energy values by LR modeling is shown in Figure 8. Both dendrograms are very similar, showing only some minor differences in the grouping inside smaller clusters. Also, the linkage distance between cluster I and other clusters was decreased using the LR predicted data set. In both dendrograms, we observe three distinct clusters as following: group I: hydrophobic carbon nanomaterials: graphene (including bilayer and trilayer), reduced graphene oxide, and amorphous carbon surfaces; group III: CNTs functionalized with charged groups of high density, as well as highly polar ZnO and ZnS nanoparticles; and group II: rest of nanomaterials, with further finer clustering in smaller groups. One can also note from the linkage distance that group I (hydrophobic materials) is more distinct from groups II and III than groups II and III between each other. By comparison of the biomolecules and nanomaterial clustering, one can note the correspondence between them. Graphene clusters interact selectively with the aromatic biomolecules (group I) due to favorable π–π interactions.^21^ Positively charged or strongly polar nanomaterials (group III) interact selectively with negatively charged biomolecules (ASP, GLU, and CYM) due to charge–charge interactions.^24,26^ One can also note that negatively charged molecules interact with surfaces of group III directly while with less polar surfaces of group II interact through intermediate water as it is shown in the insets of Figure 7. Previously, Brinkmann et al^16^ on the basis of nano-QSAR analysis of binding metabolites to various nanomaterials noted that hydrophobicity-driven interactions are important to the overall interaction strength while hydrogen bonds and other atomistic details determine differences of interactions to specific surfaces. Our results of clustering of nanomaterials are in line with these conclusions.

## Conclusion

We have analyzed data on adsorption free energies of over 30 biomolecular fragments to over 30 nanomaterial surfaces computed by classical MD simulations. We have shown that knowledge of the adsorption free energies of a small set of 3 or 5 molecules can be used to predict the adsorption free energies of other molecules. Several linear and nonlinear ML algorithms (LR, AdaBoost ensemble learning, ANN) have been tested in order to formulate the prediction model. We found that the simplest LR model provides the best result, with an average *R*^2^ score 0.88 for 10 different randomly chosen testing sets. More elaborated AdaBoost and NN models suffer from overfitting and while reproducing well the free energy of the training set, they produce a lower quality result for the testing set compared to the LR model. Overfitting may arise in more complex regression schemes with a large number of parameters such that available data are not enough to optimize the parameters, which could be counterweighted by supplying more data for training. We hypothesize that the performance of AdaBoost and NN models may be improved if simulation data for more nanosurfaces of different types, particularly charged surfaces, would be available.

We have also demonstrated that data on adsorption free energies of small biomolecular fragments can be used for clustering of nanomaterials into groups such that nanomaterials within the same group have a similar pattern of interaction with small biomolecules. This can be further translated to similar patterns of adsorption of proteins and formation of protein corona^14^ which is predictive for biological effects of nanoparticles and adverse outcomes,^2,50^ thus contributing to the grouping and read-across approach in nanotoxicity assessment.^51^ We showed that predictions of adsorption free energy by 5 chosen biomolecules produce the same result of nanomaterials grouping as clustering based on the full set of adsorption free energies. These findings can reduce the number of time-consuming atomistic simulations required to characterize bionano interactions for a new nanomaterial in order to relate it to a certain group.

The ML models presented in this work and grouping of nanomaterials are based on adsorption free energies computed in atomistic MD simulations. Besides the statistical error of the simulations, which is typically well controlled, the numerical data may be the subject of uncertainties coming from the specific force field and other limitations of the classical MD. To this point, in our work all the adsorption free energies were computed within the same methodology, including also force fields that were built according to the similar principles for the considered nanomaterials. This gives arguments that the presented in our study’s ML models of adsorption free energy predictions and grouping of nanomaterials are general and less sensitive to the specific parameters as individual adsorption free energies could be. Furthermore, one can hypothesize that since in all simulations the biomolecules were described using the same force field GAFF, which in many previous studies demonstrated good performance in comparison with experiments for small molecules in water, the statistical relationships revealed by the regressions model for a wide variety of model nanomaterials would be relevant even for experimental adsorption free energies. This assumption needs to be confirmed experimentally, but if confirmed, this would greatly facilitate experimental characterization of interactions of nanomaterials with the biological matter.

## Data Availability

Numerical data on the adsorption free energies, molecular structures and topologies of adsorbents and nanomaterials, and force field parameters (Gromacs.gro and.itp files) are available from Zenodo repository “Adsorption free energies and potentials of mean-force for interactions between amino acids, lipid fragments, and nanoparticles”, v. 2.0, https://zenodo.org/record/8297848. Workflows of computations and relevant scripts (Jupyter notebooks) are available as a part of the Supporting Information.

## Supporting Information Available

The following files are available free of charge. The Supporting Information is available free of charge at https://pubs.acs.org/doi/10.1021/acs.jcim.3c01606.

Principal components and variances of PCA analysis, *K*-means analysis of optimal number of clusters, *R*^2^ score and MAE of the regression methods, and description of the data and scripts archive (PDF)
Archive of numerical data on adsorption free energies and Jupyter Notebook scripts used in the calculations (ZIP)
