# Supplementary material for: Biomolecular Adsorption on Nanomaterials: Combining Molecular Simulations with Machine Learning
Source: J Chem Inf Model. 2024 Apr 16;64(9):3799–811. doi: 10.1021/acs.jcim.3c01606 (PMC11094735; doi:10.1021/acs.jcim.3c01606)
Supplement: Supplementary file 1 — ci3c01606_si_001.pdf [file ci3c01606_si_001.pdf]

Supporting Information

Biomolecular Adsorption on Nanomaterials: Combining Molecular Simulations with Machine Learning

Marzieh Saeedimasine, Roja Rahmani, and Alexander P. Lyubartsev*

Department of Materials and Environmental Chemistry, Stockholm University, Stockholm, SE-106 91, Sweden

E-mail: alexander.lyubartsev@mmk.su.se

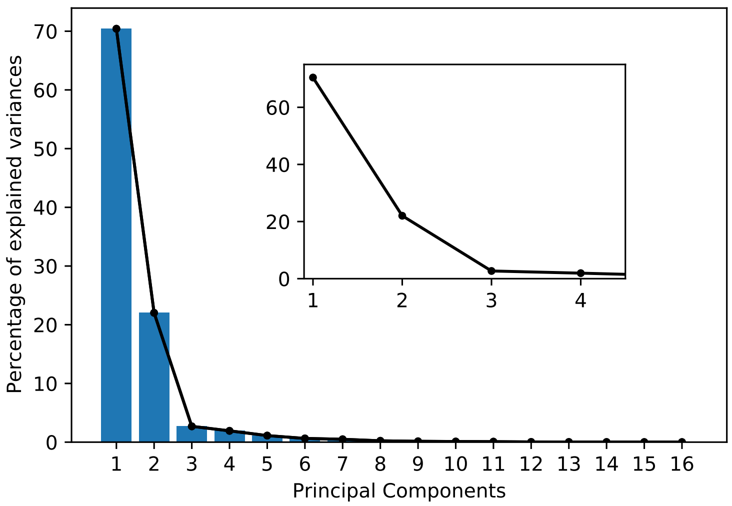

Figure S1: Percentage of explained variance along each principal component for modelling of biomolecule-surface adsorption free energy

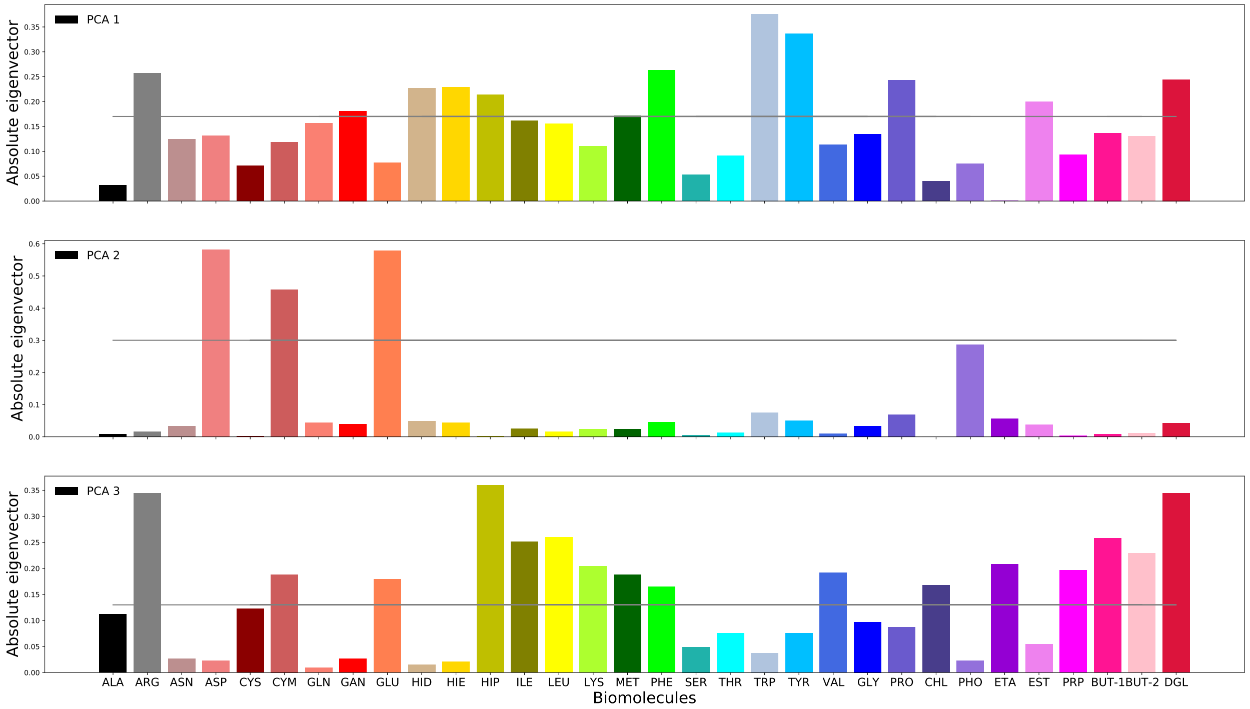

Figure S2: Absolute value of eigenvectors as a function of 32 biomolecules for three main principal components in PCA analysis

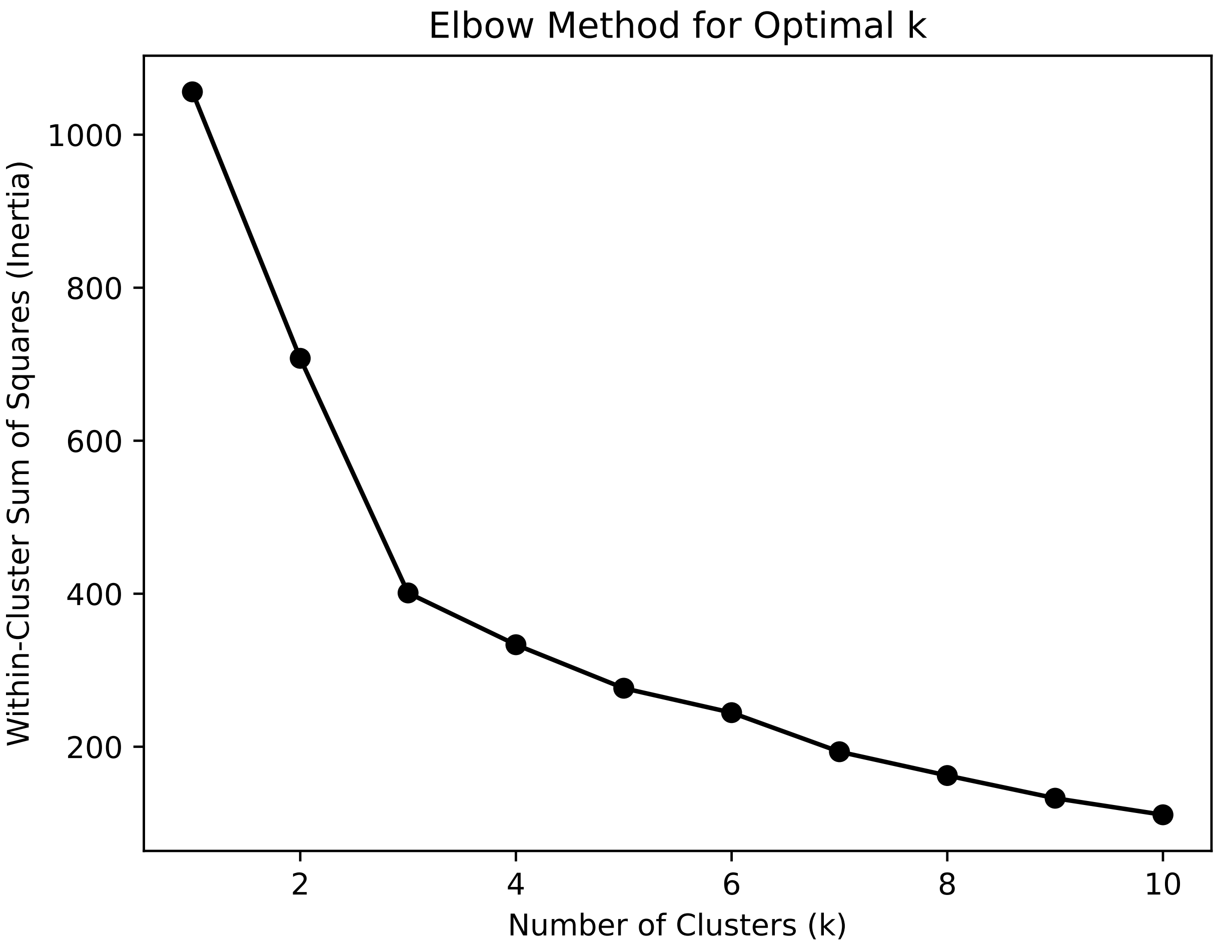

Figure S3: Determination of the optimal number of clusters for biomolecules clustering within K-means method. Shown is the within-cluster sum of squares (inertia) as a function of the number of clusters (k). The 'elbow' point in the curve, located at k=3, indicates the optimal number of clusters.

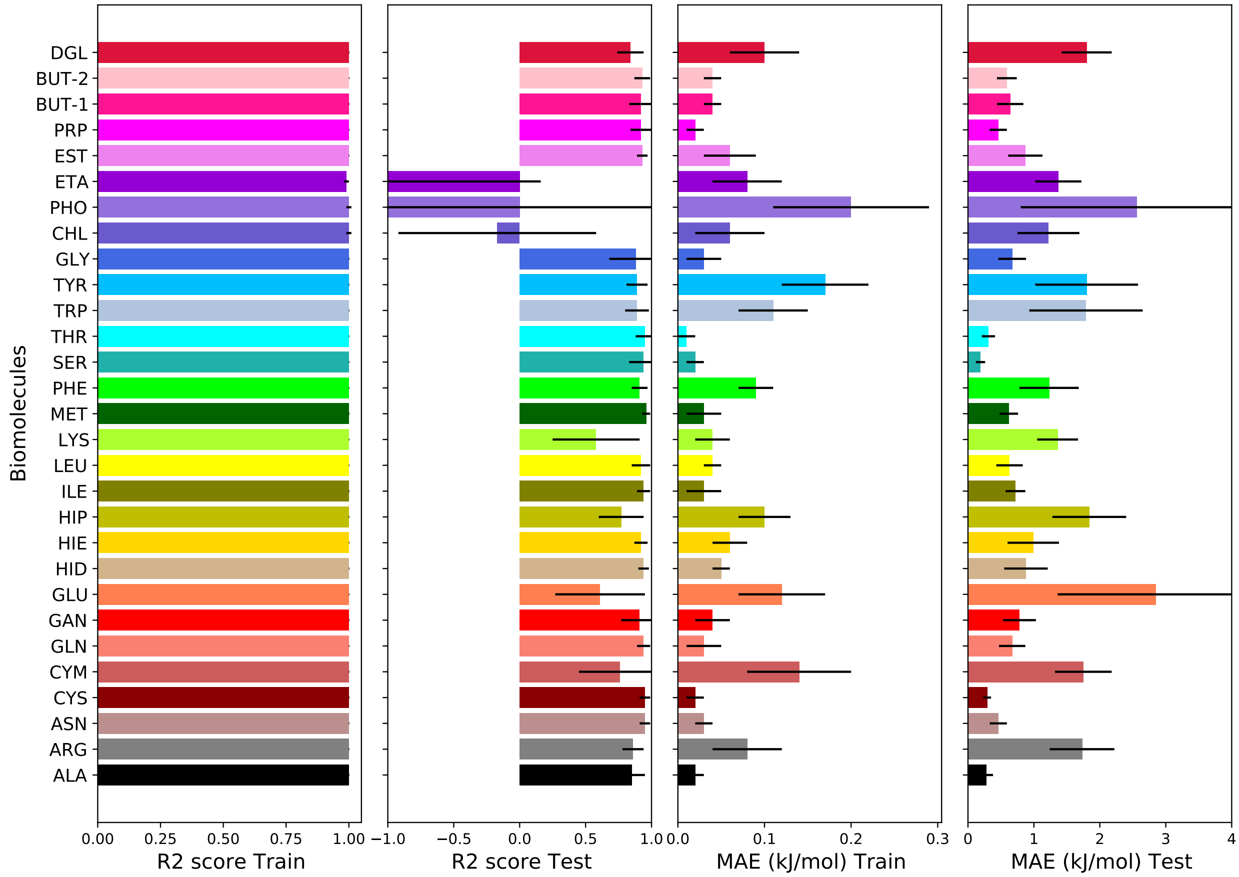

Figure S4: R^2 score and mean absolute errors for AdaBoost Regression modeling of biomolecule-surface adsorption free energies with adsorption free energies of ASP, VAL, PRO used as nanomaterials features

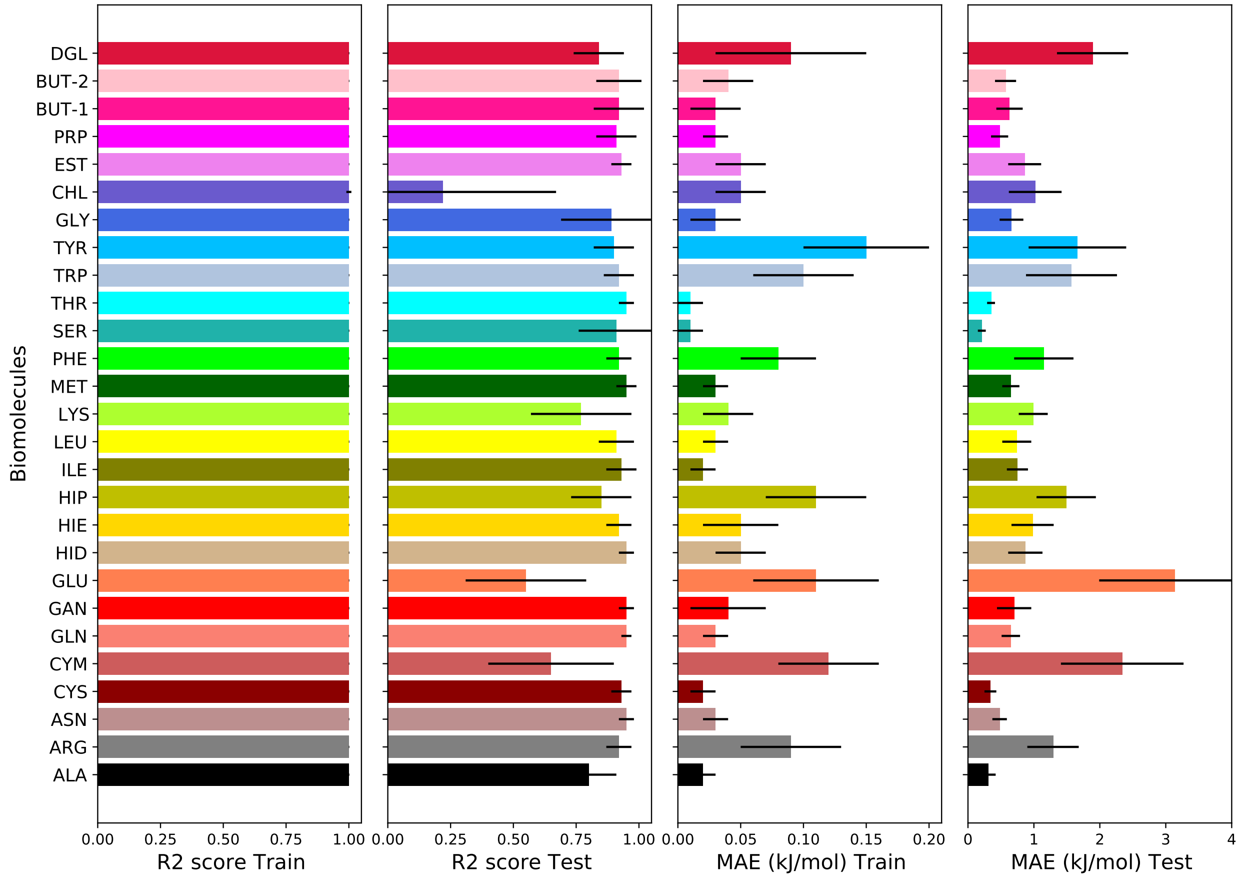

Figure S5: R^2 score and mean absolute error for AdaBoost Regression modeling of biomolecule-surface adsorption free energy with adsorption free energies of ASP, VAL, PRO, PHO and ETA used as nanomaterials features

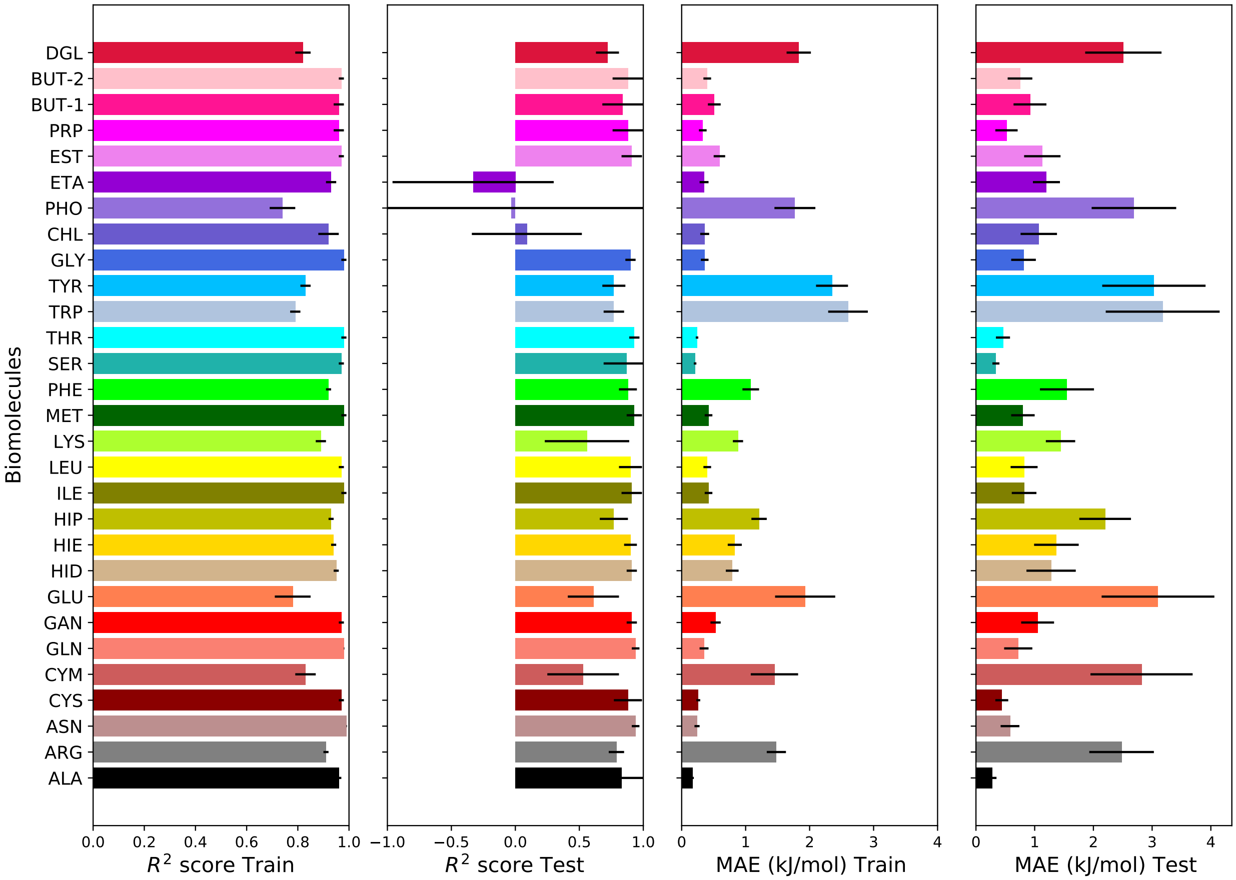

Figure S6: R^2 score and mean absolute errors for neural network modeling of biomolecules adsorption free energies with adsorption free energies of ASP, VAL, PRO used as nanomaterials features

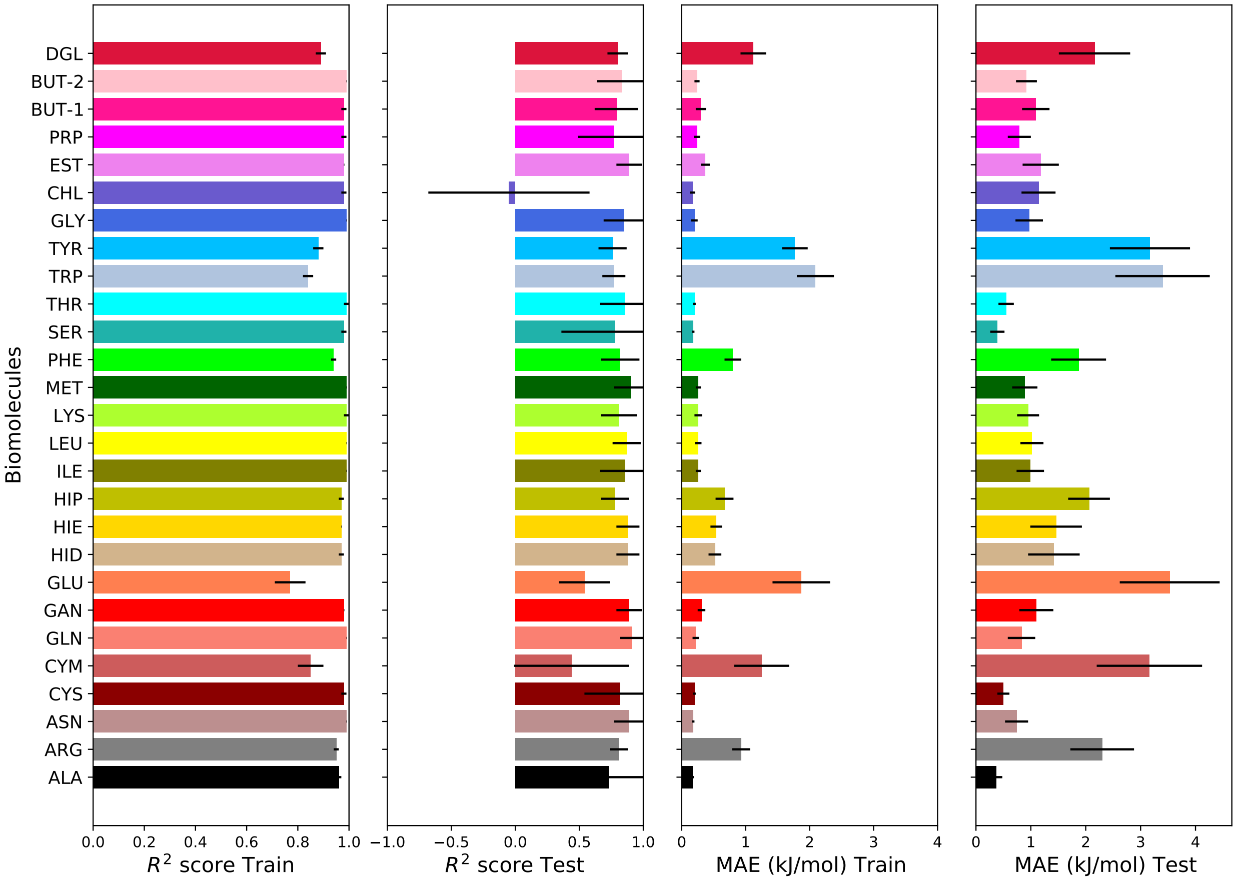

Figure S7: R^2 score and mean absolute error for neural network modeling of biomolecule-surface adsorption free energy with adsorption free energies of ASP, VAL, PRO, PHO and ETA used as nanomaterials features

Table S1: Distances of each biomolecule to the corresponding cluster center. The distance is computed as Euclidean distance in the multidimensional space: $d(x_i, c_j) = \sqrt{\sum_{k=1}^{d}(x_{i,k} - c_{j,k})^2}$ where $x_{i,k}$ is the k-th coordinate of data point i, $c_{j,k}$ is the k-th coordinate of the cluster center j to which data point i belong, and d is the dimensionality of the data space, which is 33 in our calculations.

Cluster	Biomolecule	Distance
1	ARG	9.74
1	DGL	17.94
1	EST	14.81
1	GAN	16.59
1	HID	7.43
1	HIE	7.04
1	HIP	12.23
1	PHE	9.32
1	PRO	6.71
1	TRP	24.75
1	TYR	18.97
2	ALA	17.69
2	ASN	6.22
2	BUT-1	9.19
2	BUT-2	7.25
2	CHL	15.30
2	CYS	9.09
2	ETA	22.95
2	GLN	12.11
2	GLY	11.50
2	ILE	12.53
2	LEU	11.54
2	LYS	11.31
2	MET	13.83
2	PHO	35.89
2	PRP	7.29
2	SER	12.83
2	THR	5.31
2	VAL	5.17
3	ASP	12.15
3	CYM	17.96
3	GLU	17.32

Table S2: Coefficients and intercept values of Linear Regression model in which adsorption free energies of ASP, VAL, PRO, PHO and ETA are used as nanomaterials features for prediction of free energies of other biomolecules according to: $G_{ads,i} = \sum_j a_{ij} G_{ads,j}$, where j is running over the five selected biomolecules while i labels all other biomolecules.

Molecule	ASP	VAL	PRO	ETA	PHO	Intercept
ALA	-0.001	0.542	-0.118	0.008	-0.013	0.104
ARG	-0.041	-0.632	1.340	0.493	-0.090	0.057
ASN	-0.010	0.056	0.473	-0.041	0.014	0.263
CYS	0.001	0.641	-0.003	0.006	-0.010	0.070
CYM	0.726	0.704	-0.477	0.266	0.175	-0.475
GLN	-0.011	0.140	0.563	-0.057	0.021	0.320
GAN	-0.034	0.093	0.662	0.014	0.038	0.048
GLU	0.935	-0.104	0.251	0.083	-0.021	0.428
HID	-0.059	0.105	0.825	-0.114	0.051	-0.132
HIE	-0.054	0.101	0.843	-0.053	0.028	-0.055
HIP	-0.046	-0.702	1.191	0.545	-0.097	-0.113
ILE	-0.003	1.217	0.085	-0.029	0.025	-0.186
LEU	0.001	1.344	0.004	0.041	0.016	-0.092
LYS	0.017	0.110	0.436	0.817	-0.068	0.396
MET	-0.019	0.961	0.241	-0.064	0.017	-0.140
PHE	-0.057	0.867	0.622	-0.088	0.059	0.096
SER	0.002	0.300	0.084	0.001	-0.013	0.369
THR	0.003	0.477	0.157	0.009	-0.009	0.360
TRP	-0.097	0.398	1.256	-0.134	0.081	-0.042
TYR	-0.139	0.456	1.021	-0.076	0.148	-1.015
GLY	0.001	-0.109	0.610	0.107	-0.012	0.019
CHL	0.048	-0.311	0.390	0.136	-0.142	0.354
EST	-0.053	0.365	0.603	-0.111	0.043	-0.057
PRP	-0.017	0.789	0.005	-0.101	-0.000	-0.102
BUT-1	-0.019	1.088	0.036	-0.090	0.005	-0.304
BUT-2	-0.022	0.953	0.073	-0.097	0.011	-0.049
DGL	0.014	-0.413	1.189	0.858	0.023	-1.161

Test with 30% of nanomaterials excluded from the clustering stage

In order to test that there are now data leaking to the results of the testing set from the initial clustering of biomolecules which is based on the whole data set, we repeated the procedure starting from the reduced set of nanomaterials in which 30% of nanomaterials were randomly excluded. Result of the hierarchical agglomerative clustering of biomolecules carried out for this reduced set of nanomaterials, shown in Figure S8, is very similar to the clustering carried out for the full set, with the most noticeable difference in that PHO residue moved to the group III with other negatively charged molecules. Still, the same molecules: VAL, PRO and ASP were identified as closest to the cluster centers. Adsorption free energies of these 3 molecules were used as features in training of the LR model on the same reduced set on nanomaterials as used in the hierarchical clustering while remaining 30% of nanomaterials were used as a testing set. Results for R^2 score and MAE are shown in Fig. S9. When adsorption free energies of "outliners" (with negative R^2 score) PHO and CHL were added to the set of features, the LR model with 5 features again showed R^2 score and MAE (Fig. S10) similar to the case when data for all nanomaterials were used in the agglomerative clustering (see the main text), with the average R^2 score 0.86 and MAE 0.93 kJ/mol.

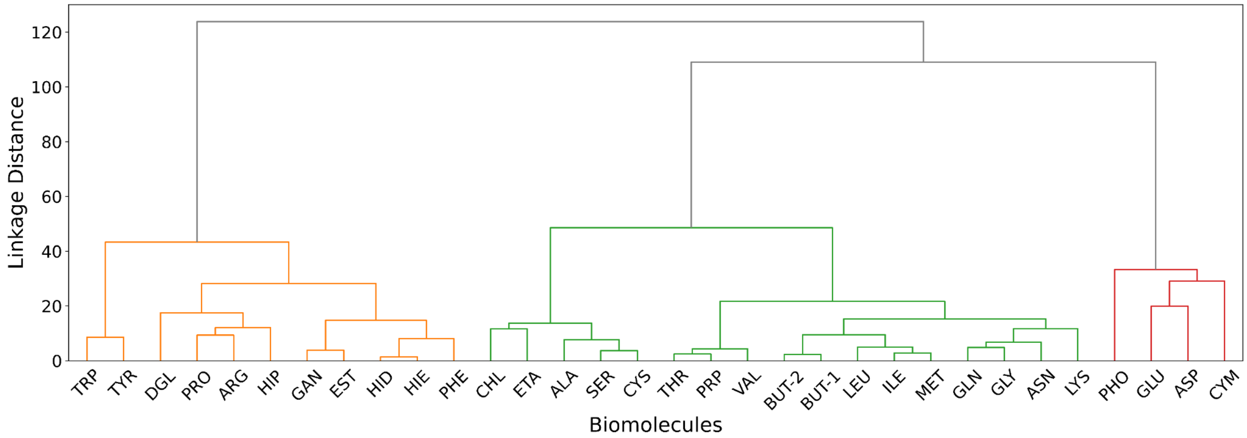

Figure S8: Dendrogram of biomolecules agglomerative clustering based on the reduced data set of 70% of randomly selected nanomaterials.

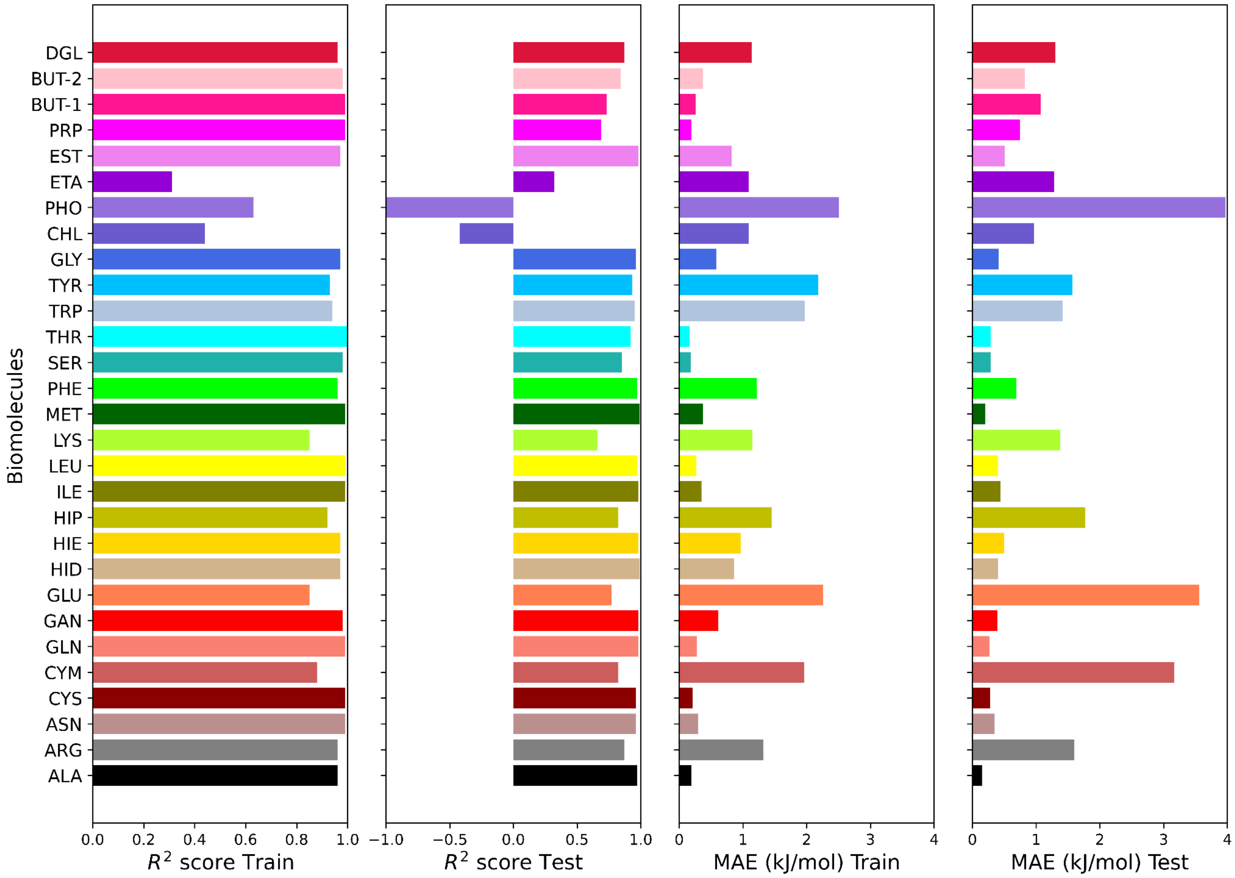

Figure S9: R^2 score and mean absolute error for linear regression modeling of biomolecule-surface adsorption free energy with adsorption free energies of ASP, VAL, PRO biomolecules as nanomaterials features, for the training set of nanomaterials (the same 70% of nanomaterials which are used in agglomerative clustering) and testing set (other 30% of nanomaterials)

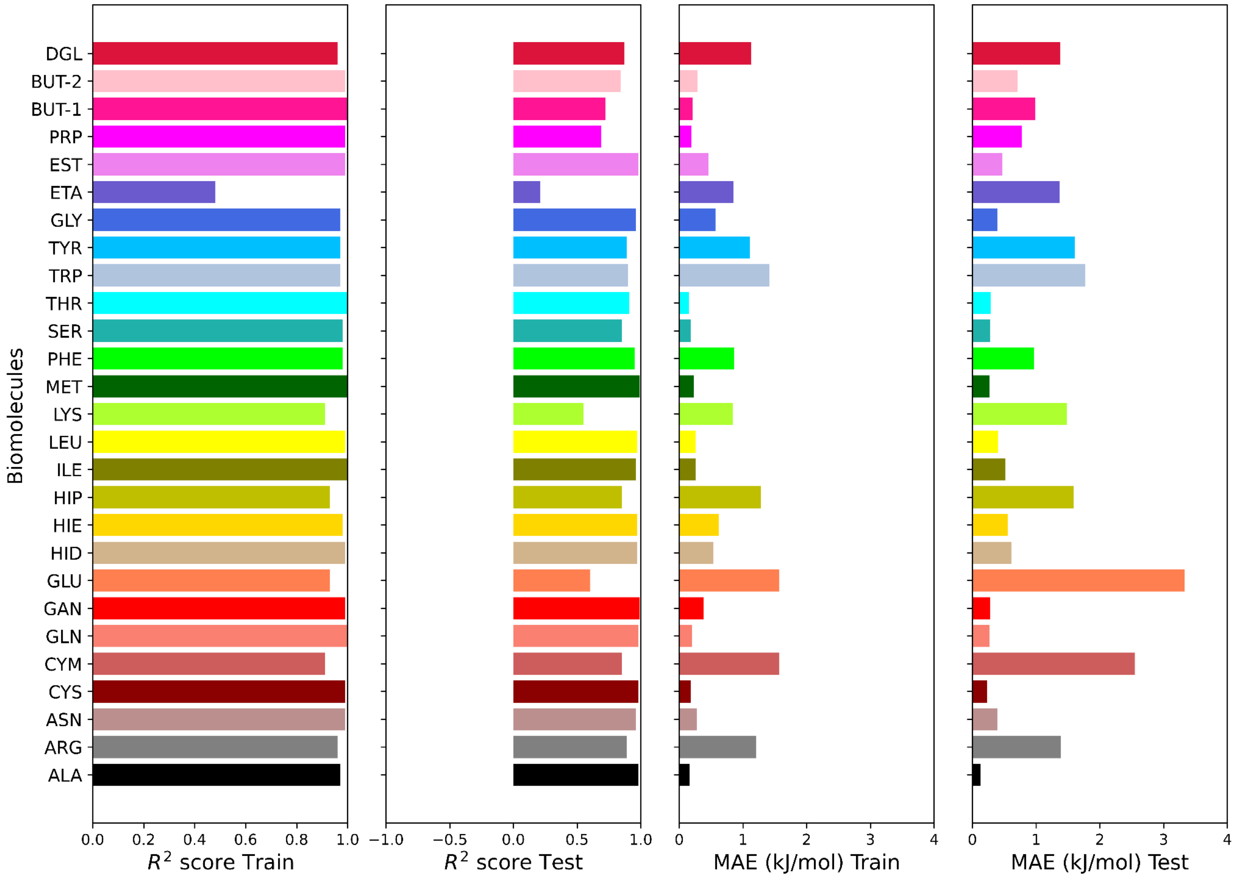

Figure S10: R^2 score and mean absolute error for linear regression modeling of biomolecule-surface adsorption free energy by adding free energies of PHO and ETA biomolecules (besides ASP, VAL, PRO) as nanomaterials features, for the training set of nanomaterials (the same 70% of nanomaterials which are used in agglomerative clustering) and testing set (other 30% of nanomaterials)

Description of the data and scripts archive

Data on adsorption free energies of small biomolecules to different nanomaterials, and Jupyther Notebook scripts implementing machine learning methods described in the paper, are collected in archive `Data_and_scripts.zip` provided as a part of the Supplementary Information.

The archive is organazed as follows.

The top directory contains brief description of the archive, licensing information, and full data set of adsorption free energies of 32 small biomolecules to each of 33 nanomaterials considered in the paper. The data are provided in a plain text format with space-separated fields (`Data_and_scripts.txt` file), and in the excel .xlsx format. For each combination biomolecule-nanosurface, the value of the adsorption free energy is given together with estimated upper and lower error boundaries.

The scripts, accessible by the Jupyther Notebook, are collected in directory `scripts`. The scripts describe workflow of the computations and results presented and discussed in the main text of the paper. The following scripts are provided:

- `PCA.ipynb`: Principal Component Analysis for Linear Dimensionality Reduction and Eigenvectors analysis along the original features.
- `Clustering.ipynb`: Biomolecules Agglomerative Clustering and K means Clustering; Nanomaterials Agglomerative Clustering using both full data set and predicted adsorption free energies data set
- `LR-ML.ipynb`: Linear Regression modelling of Nanomaterial-Biomolecule adsorption free energy
- `AdaBoostRegressor-ML.ipynb`: AdaBoostRegressor modelling using DecisionTreeRegressor as week learner for Nanomaterial-Biomolecule adsorption free energy

- `ANN-ML.ipynb`: Neural network modelling of Nanomaterial-Biomolecule adsorption free energy
